# Observations on the Epidemic Yellow Fever of Natchez, and of the South-west

**Published:** 1842-09

**Authors:** John W. Monette

**Affiliations:** Washington, Mississippi


### WESTERN JOURNAL

OF

### MEDICINE AND SURGERY.

SEPTEMBER, 1842.

ART. I.—*Observations on the epidemic Yellow Fever of Natchez, and of the South-west.* By JOHN W. MONETTE, M. D., of, Washington, Mississippi.

(*Concluded.*)

Admitting the correctness of the positions which we have assumed, the question which naturally presents itself is this: *How shall New Orleans and the towns on the lower Mississippi be protected from* EPIDEMIC *yellow fever?*

We answer, they may be protected from devastating *epidemics* by judicious quarantine regulations, properly enforced, near the mouth of the Mississippi, or at any proper distance below the city. These regulations should have for their object the prevention of any direct intercourse with Vera Cruz, and Havana, or any other West India port, during the time that such port is the seat of epidemic yellow fever. This would necessarily exclude shipping from Vera Cruz generally from and after the first of June; and from Havana, from and after the first of July, varying according to the periods at which those ports become imminently infected. This prohibition should be enforced for two months or more, until the epidemic subsides in such ports.

The quarantine regulations should also embrace some provision for reducing the number of transient, unacclimated strangers or northerners, from and after the middle of July, and also for prohibiting the introduction of foreign emigrants into the city between the months of June and October; and, when yellow fever is virulent in the West Indies, the prohibition should be enforced until the last day of October. The danger of any epidemic is always augmented by the influx of northern foreigners during its prevalence. This is well known in New Orleans; and the city press does not fail to warn them of their danger, and vainly to deprecate their arrival.* The civil authorities should coerce their exclusion, if necessary, during an epidemic, or during the apprehension of one. An epidemic cannot become virulent, and extend its influence rapidly among the resident population, without the aid of a large number of northern strangers and foreigners, who are unacclimated; and during an epidemic they are the fuel which keeps up the fire.

If the disease in its epidemic form can be excluded from New Orleans, every interior town in the south-west, having a direct commercial trade with, and dependence upon that city through the lower Mississippi, will most assuredly remain free from this pestilence; and their citizens may rest secure from the apprehension of danger from that quarter. All quarantine restrictions on the river above New Orleans will be unne-

---

* The following extract from the Louisiana Advertiser, of October, 1839, will show the general feeling on this subject, viz.:

"*Food for yellow fever.*—The barque Eleanor, which arrived yesterday morning from Havre, brought to the city sixty steerage passengers, chiefly Germans: they are full-blooded, fresh, and healthy—*inviting subjects for yellow fever*. We looked on them as among the *doomed;* and sorrowed to think that such robust and strong-constituted individuals, should be grappled by so fierce an enemy."

cessary; and the interior commerce of the city, with the valley of the Mississippi, will be constant and uninterrupted.

This brings us to speak more particularly on the subject of quarantine regulations, adapted for the protection of New Orleans and other towns on the lower Mississippi.

### QUARANTINE.

There are in all countries, and especially in commercial cities, men whose pecuniary interests conflict with wholesome regulations for the welfare of the community at large; men whose feelings and views of things mislead their judgments relative to the general good of society. In nothing is this principle more clearly elucidated, than in the opposition usually made to all temporary restriction upon commerce, by sanatory quarantine regulations.

Notwithstanding the apparent efforts, sustained by the commercial interests, and the weight of medical authority in the United States, to abolish all quarantine regulations designed for the protection of our commercial ports, the facts are continually staring us in the face, that *there are certain diseases*, peculiar to tropical ports, or to certain cities and regions of the civilised world, which are more or less communicable from one person to another; and which, when epidemic in any city, may, by a favorable combination of circumstances, which are not well understood, be communicated and disseminated in other towns and ports, which were previously free from any such disease. The two most prominent diseases of this character, are the *plague* of the eastern continent, and the *yellow fever* of the western, not inaptly termed the western plague. These diseases, when communicated from one port to another, are supposed to be imported solely through the agency of ships and commercial intercourse. When one port or city is more favored by nature than another, either in its location, latitude, or any other circumstance, which renders it exempt from diseases which are endemic in others, it becomes the natural right of the inhabitants, and the special duty of the municipal authorities of such favored port or city, to protect themselves against all the possibilities of introducing a pestilential disease.

As such diseases are caused by some invisible, uniform, and mysterious influence, which manifests itself only in those persons who are exposed to its action; and as the same disease, when produced in a healthy place, is the result of a similar exposure to the influence of a portion of the same atmospheric agent, which may have been, and doubtless has often been carried from infected ports, in ships and other articles freighted in them, it is the dictate of reason and prudence, to *interdict* temporarily, that kind of intercourse with such infected port, which affords the greatest probability of introducing the aerial poison. This temporary interdiction of commercial intercourse with cities which are the seats of epidemic pestilential diseases, has been denominated a quarantine restriction.

The older quarantine regulations, from some superstitious veneration for the number forty, required vessels from such infected ports to remain out of port for forty days: hence the name. Such restrictions, under one form or another, have been enforced from the earliest history of the commercial world; and the world, by common consent, has approved the same. But with the nineteenth century chiefly, and chiefly in the United States, the doctrine has sprung up, and has been propagated, which teaches that the light of science and the march of intellect, at this late day, deem it expedient to expose populous cities to the ravages of pestilence, rather than impose any wholesome restrictions upon the interests of the few, to avert such calamities.

Such regulations have always been opposed by the commercial interests in any port, because it does not ostensibly administer to the pecuniary advantage of that class of individuals. At length many of the learned of the medical profession have been brought over to sustain this opposition, which, at this time, has become somewhat *popular* in the United States. This circumstance alone is calculated to draw a formidable array of learning and talent against any cause, however sound its positions.

Besides the professions of philanthrophy, benevolence, charity and humanity, as the motives which prompt the opposition to judicious quarantine restrictions, many have taken the ground that the disease—the pestilence to be excluded by such means, is *endemic in our ports;* the product of local causes which abound in all our towns, independent of, and unconnected with foreign intercourse: and that consequently it is not to be excluded by such restrictions against foreign importations. Through the name and influence of Dr. Rush and his coadjutors, backed by the allies of the commercial and importing classes, this salutary protection to the population of some of our most important seaports, had well nigh been abolished. Yet they have passed from the stage of action, and their influence, losing its power over the judgments of men, ceases to mislead on this important subject.

New York, Philadelphia and Baltimore, as if for the purpose of testing the doctrine of foreign importation of yellow fever, at the expense of thousands of their citizens, refused to adopt any decided restrictions on the West India commerce until about the year 1822, when the experiment had been fully made. Since that time, the counterpart, and by far the most salutary part of the experiment, has been in operation.

Since the year 1822, the quarantine restrictions enforced at New York and Philadelphia have protected those ports, and for twenty years they have escaped all pestilent epidemics. Yet before these restrictions were enforced, they had been liable to these visitations, no less than six times in 20 years, from 1790 to 1810.

The southern ports of Charleston, Savannah, Mobile, and New Orleans, where quarantine regulations are discarded, are almost annually ravaged by this West India plague. Will not the same regulations, strictly enforced in these southern ports, be followed by the same salutary results?

It will be remembered that we have already set forth the manner in which vessels and steamboats become infected, and instrumental in the introduction of yellow fever into a healthy port, to which we refer the reader.

*How should a quarantine be enforced below New Orleans?* We answer, 1st. establish a quarantine ground near Fort Jackson or Fort St. Philip, which shall effectually command the river. From the time that yellow fever has assumed its epidemic form in Vera Cruz, Havana, or any other place, cause any and every vessel from such port, to remain below at the quarantine ground: there let her cargo be discharged, and properly ventilated for 72 hours, at least, day and night, before it is allowed to be taken up to the city: the importing vessel should not enter the port of the city until after frost. At the same time, vessels from healthy ports, which have not touched at any infected port, might be permitted to ascend and discharge their freight at the wharves of the city—provided their crews are perfectly free from disease.

2. Erect a large, airy, brick hospital, at the quarantine ground, for the reception of seamen and others, laboring under yellow-fever, from vessels detained. The buildings should be comprised in an extended row of one-story houses, with rooms about 20 feet square; each room to accommodate four patients.

3. Prohibit, during the quarantine, the entrance of foreign emigrants, or strangers who have touched at any infected port within ten days previously.

4. The Charity hospital, for the reception of the indigent sick of the city, should be located in the suburbs, at least half a mile from the populous part of the city.

It can be well established that whenever yellow fever has prevailed in any port of the United States or in any port of Europe, it was always at a time when a direct intercourse had been kept up between such port and the West Indies where yellow fever is endemic. Whenever it has been epidemic in Lisbon, Cadiz, or Gibralter, it was immediately preceded by an unusual intercourse from the West Indies to those ports. This was true, especially, from 1804 to 1814, when large armaments and fleets were constantly arriving from Havana, Vera Cruz, and other tropical ports, during the European wars with Bonaparte.

The same is true of ports in the United States. We have already shown that yellow fever is not epidemic in them except when the intercourse with the West Indies is direct and uninterrupted. This point can be established by a reference to every instance in which it has prevailed as an epidemic in our cities. Dr. Hosack observes, that "during the years in which our commerce with the West India ports was interdicted by the embargo (before the year 1812), and during the subsequent years of war with Great Britain, when our communication with her possessions in the West Indies, and other West India ports, was entirely suspended, *the cities and towns along our whole coast were exempt from the fever of the tropics.* A recurrence to meteorological observations, for that period, will show that the mercury frequently ranged higher, than in those years when the yellow fever prevailed in our cities; and when the general constitution of the atmosphere, which was favorable to the generation of this malignant form of fever, provided domestic causes could engender it, pervaded our country as much as at other times, when yellow fever had prevailed extensively. The local causes in our sea-ports, such as exhalations from our ships, market places, privies, and new-made grounds, remained the same, and in some cases worse than when the yellow fever had prevailed in other years. Yet, unfortunately for the doctrine of domestic origin of yellow fever, the health of our cities remained undisturbed by the deadly visitor."* The same was true of all our ports during the war of the revolution, from 1776 to 1785. But so soon as peace was confirmed, and a brisk trade was carried on with the West Indies, the yellow fever began to prevail in all our most important ports, from 1790; and it prevailed extensively in some of the principal ports, nearly every other year, until the embargo in 1808 and the war of 1812 so interrupted the commercial intercourse with the West Indies, that epidemic yellow fever was not known in the United States until the year 1817, when a constant commercial intercourse with the West Indies had become again established.

* See Hosack's Medical Police.

Since that time the intercourse with tropical America has been uninterrupted, and yellow fever as an epidemic has not failed to visit our most important ports, almost every other summer, up to this time.

The great practical error and impolicy of the *domestic origin* of the yellow fever, is this: viz. it leads people to disregard the proper precautions against its dissemination through a dense population; it indirectly encourages its diffusion among a healthy population, by encouraging those habits of careless intercourse by which the limits of the infection is certainly extended; while every measure which tends to circumscribe its limits are neglected or disregarded. Under the belief that it cannot be imported, or even communicated, directly or indirectly (because it is local and indigenous in its origin), many persons are induced to expose themselves to infected ships, houses, and the infected districts of a city, and thereby become instrumental in carrying the disease in their own persons into healthy parts of the cities and towns. This is often the case with persons who have no definite ideas of any of the laws of infection and contagion, and are often blindly impressed with the belief that the disease is "not catching." It leads also to a culpable carelessness with the sick, by indirectly encouraging the accumulation of the morbid effluvia in their rooms, and the accumulation of morbid secretions about their beds and chambers; for, as is very naturally inferred, if no effluvia or morbific exhalation is given off by the patient, why the necessity of cleanliness and ventilation? It encourages a disregard of all sanatory cordons which may be established to cut off the intercourse of the healthy population from the infected vessels, houses, or districts. It is to be regretted that medical men, the proper conservators of health, should be foremost in inculcating doctrines which tend indirectly to lead on these malignant epidemics into our cities.

Medical men are often led by theory, or the force of education, to disregard important facts in these epidemics; or to bend them to suit a preconceived theory: but the intelligent mass of the people have neither theory nor the bias of education to mislead them, and they generally judge correctly upon the facts which present themselves. It is a well known fact that the mass of the intelligent population in our southern ports, where yellow fever is often epidemic, are involuntarily led to the conviction, that it is an imported, and, under some circumstances, a communicable disease. They know that ships and steamboats do sometimes become strongly infected while the resident population is healthy. This conviction is produced in their minds, without any desire to sustain any theory, or from any pride of opinion. As the Abbe Du Pratt has remarked, "there is often *a blind impulse in public opinion which arrives by a more speedy and certain method at the truth*, than all the reasonings of philosophy."*

Those who oppose all quarantine restrictions, urge the plea that the commercial interests are deeply embarrassed, and the general prosperity of the cities interrupted. These we conceive to be false positions. We contend, on the contrary, that the health, prosperity and pecuniary interests of all classes are promoted by proper quarantine regulations. For example, let us take New Orleans and Natchez, which might be protected from yellow fever epidemics by judicious quarantine restrictions.

When Natchez is the seat of epidemic yellow fever, the whole commercial business of the place may be said to be prostrated for that time; all mechanical business and trade is stagnant for two or three months, and the ordinary income of at least five hundred families† is suspended, and their daily expenses increased fourforld, by flight, sickness, abandonment of property and new expenses incurred by procuring a temporary residence out of the city. In this we have not included the permanent loss which the community sustains in the death of two or three hundred of the most enterprising and useful citizens, at each visitation, exclusive of an incalculable amount of suffering, privation and bereavement which are but

---

* See Townsend on Yellow Fever of New York, p. 62.

† The entire population of Natchez during the summer is 3500.

partially indicated by the emblems and weeds of mourning which shroud the community for months afterwards. All this is avoided and escaped during the summers when yellow fever does not visit the city; and with incalculable advantage to the dependent classes.

Whatever may be the foreign trade of New Orleans, it is almost entirely suspended during the prevalence of an epidemic in the city; and, what is of far more importance to the prosperity of the city, the whole inland commerce of the valley of the Mississippi, with a population of more than four millions, is likewise suspended. Besides this, nearly five thousand of the resident population annually leave the city in anticipated fear of an epidemic, and an equal number leave on the certain appearance of the unwelcome visitor. In addition to this, the city suffers a permanent loss by the death of not less than two thousand or twenty-five hundred souls, during each epidemic; and among this number may be reckoned about five hundred enterprising and useful citizens forever lost. We might add, without taking into the estimate the amount of mental and physical suffering, that the charge, responsibility, and expense of providing for, nursing, and interring more than one thousand destitute strangers, is thrown upon the city at each epidemic visitation.

All this may be avoided by excluding yellow fever from the city; and the enforcement of a judicious quarantine near the Balize, and on any other commercial avenue for three months at most, during the prevalence of yellow fever in the West Indies, affords the only prospect of accomplishing this desirable result. New Orleans may be, and at length will be released from this annual pest, if her citizens will it to be done.

The plea of humanity, of philanthrophy, in this case so often preferred, as an argument against quarantine restrictions, with apparent sincerity, is perfectly gratuitous. If we admit the position that yellow fever is local in its origin, and has no tendency to spread among a dense population, and that the multiplication of cases does not tend still further to contaminate the air; and that quarantine restrictions encourage the abandonment and entire neglect of the sick, it then would appear a very plausible argument, *ad captandum.* But denying all these assumptions, we contend that we are entitled to the whole benefit of the argument on the score of humanity and philanthrophy. We urge that it is not local and that it can be excluded from our ports; and by so doing we should be the means of preserving the lives of thousands of persons annually in the United States; and we should in like manner protect our citizens from all the privations and sufferings incident to these epidemics, and the concomitant stagnation of every species of trade and business upon which the poor depend for daily subsistence. We *prevent* the misery and wretchedness which others would in vain attempt to *mitigate*, after they have aided in bringing it about.

We have devoted none of our remarks to the treatment of this disease. All the experience of the medical profession in the United States, for fifty years, and all the researches of science, and all the *post mortem* examinations of organs and tissues, have tended to throw no light on the treatment. Under all modes of treatment, consistent with the various theories framed for its cure, the disease in its most malignant type sweeps off into the grave at least one half of its victims, and with as much celerity and certainty now as it did when first introduced into our ports fifty years ago. The milder cases which occur in all these epidemics, will recover under any judicious mode of treatment much more readily than severe cases of common remittent fever. Many of the mildest cases will recover almost without any medical treatment. But the severe cases generally terminate in death, unchanged by any mode of therapeutic treatment. Hence we doubt not that it is much more important to the interests of our cities and ports, to direct attention to *preventing* these epidemics, than to discover the best modes of cure after they have been invited among us.

### CONCLUSIONS.

If the views advanced in the preceding pages be correct, we are sustained in the following as legitimate deductions:

I. That the miasm or gaseous matter which is essential to the production of epidemic yellow fever, is generated only while the extreme temperature in the shade is at least up to 88° of Fahrenheit; and that so long as there is sufficient agitation and change of the air by winds, it will not accumulate in sufficient quantity to produce yellow fever as an epidemic: that when sufficient miasm is produced and accumulated, the malarious combination, which likewise requires several days of calm, sultry weather, will proceed at a still lower temperature.

II. That the miasm of yellow fever, is, *per se*, in a pure atmosphere, probably innoxious; but acquires active morbific properties by combining with sultry, hot air, which has been exhausted by respiration, and charged with human exhalations; and that it then becomes *malaria*, or infectious air, which is an active predisposing cause, as well as a proper "*nidus*" for the reception and evolution of the infection of yellow fever.

III. That this malarious condition of the local atmosphere of any city, or portion of a city, may be so concentrated as to produce a strong predisposition to yellow fever in many of the inhabitants, without actual disease, until after a few cases have been excited into action by highly exciting causes, when *infection* is generated, and speedily the malarious district becomes the infected district: which result would have been prevented by a storm or change of weather previous to those cases.

IV. That when the malarious combination is sufficiently concentrated for this purpose, a large quantity of *infected air*, brought from an infected district, or a large number of cases of yellow fever introduced from another point, will convert that malaria into infected air, and produce an epidemic likewise.

V. That consequently, although yellow fever may to a certain extent be called a disease of local origin, and depend upon a local atmospheric constitution, or contamination, for its extension, yet it may, under some circumstances, be carried from one city to another, and there propagated.

VI. That accordingly epidemic yellow fever may be averted sometimes by one or all of the following measures, enforced at a time, when, according to the principles herein set forth, the malaria is forming rapidly; viz:

1. By a dispersion of the greater portion of the citizens to the country.

2. By removing from the city, and especially from the districts usually infected, all *strangers*, or those who have not become acclimated by a residence of two or three years, and who would of course be the first attacked.

3. By prohibiting the introduction from foreign places of infected air, or *fomites*, or patients laboring under yellow fever during the prevalence of malarious accumulations.

VII. That malaria accumulates most in those parts of cities and towns, where the population is most crowded, and where, from the situation of the ground, or the nature of the buildings and enclosures, the local atmosphere becomes most stagnant; and such accumulations of malaria in the United States take place chiefly in the months of July, August, and September.

VIII. That when yellow fever rages like a pestilence in any city or town, the suburbs, the immediate vicinity, and the whole surrounding country, are entirely free from such infected air, and from all similar disease. Of course the epidemic yellow fever cannot be the result, solely, of "a general atmospheric constitution"—but is the result of some peculiar local contamination of the city atmosphere.

IX. That this local contamination, or infection, *is not* the product of the city filth usually found in streets, alleys, and sewers, and about the wharves; nor is it the product of the decomposition of animal or vegetable matters, under any circumstances.

X. That when this contamination or infection is once formed in the local atmosphere, none of the disinfecting agents heretofore used, have any virtue in neutralising the infection, or in arresting the progress of an epidemic. Cold, or a reduced temperature, is the only known destroyer of the infection of yellow fever.

XI. When an epidemic has once begun, the only safety to the unacclimated is speedy flight to the country, where the air is pure. When the population of an infected town has fled in great numbers to any healthy town, carrying with them large quantities of clothes, beds, bedding, blankets and porous articles of merchandise, there is great danger to be apprehended, lest the fomites should there generate a new local infection equally virulent with that from which they had escaped. These articles, when infected, are liable to become the most virulent sources of infection, and are purified only by frost or cold weather.

XII. That all steamboats, flat-boats and vessels which remain at the wharves for many days, in an infected district, discharging and receiving freight from the infected district, do often become as thoroughly infected as the houses in that part of the town, and finally become as capable of imparting the disease to unacclimated strangers, as the air in the city houses would, under similar circumstances.

XIII. Consequently such vessels from an infected port, should be prohibited from lying at the wharves of a healthy town, and from discharging their freight or sending into such town yellow fever patients, or in any wise holding intercourse with the population of that town during the months of July, August or September.

XIV. That the cause of humanity, both in relation to the sick and the well, and especially to the latter, requires that all hospitals provided for the reception of boatmen, sailors, and all indigent poor, during an epidemic yellow fever, should be located beyond the limits of any city or town, and that the free and promiscuous intercourse of the citizens with such hospital should be interdicted.

XV. That all empty and unoccupied flat-boats at the wharves should be removed early in summer, as they contain a large amount of stagnant atmosphere constantly acted on by the intense rays of the sun, and they may thus become reservoirs of what we have termed *miasm* and *malaria.*

The following *facts* relative to yellow fever in its *epidemic form*, appear to be well established by experience and observation, especially as regards the United States.

1. Foreigners and northern strangers are often attacked with yellow fever in the southern ports of the United States, as well as in the West Indies, when the whole resident or acclimated population is healthy; and the former are the principal victims during any yellow-fever epidemic.*

2. It is extremely rare that any person is attacked a second time by yellow fever. The first attack produces such a change in the constitution that those who recover are generally forever afterwards exempt from its attack, while they reside in a southern latitude.†

3. In the most virulent epidemics, at least *one half* of those attacked by yellow fever die; when less than that proportion is reported, we may believe many cases have been enumerated as yellow fever which *are not yellow fever;* or that the disease assumes an uncommonly mild form.

4. When an infected district is once formed, the infection spreads slowly and gradually from one or more points, or radiating centres, until it pervades the principal parts of such city or port. In its progress it advances steadily, regardless of promenades, burying-grounds, compact or open squares; over sewers, cist-pools, clean or filthy streets, and in wet or dry weather; it pursues its retreating victims to their farthest retreat, provided they have contracted the seeds of the disease, or breathed the concentrated infection.

5. During an epidemic the virulence of the infection is *more active by day* than at night, and exposure in the infected dis-

---

* See Townsend on the Yellow Fever of New York, pp. 249–252.
† Vide Ibidem, pp. 247–9.

trict is more dangerous at noonday than at night. Night-watches are more exempt, *ceteris paribus*, than the day-watch, from the attacks of the disease.*

6. Common city filth *has no agency whatever* in generating yellow fever epidemics; on the contrary, it is calculated to absorb or neutralise the infection.† Scavengers and grave-diggers have always been more exempt from attacks of this disease than others.

7. Lime as a *prophylactic*, or a *disinfecting agent*, is *useless if not prejudicial.* Those engaged in spreading lime in cities have been more frequently attacked by the disease than the scavengers. In New York in 1822, the yellow fever spread much more rapidly in Lombardy and Cheapside streets, *after* lime had been spread in them, than previously.

8. Next to blankets, feather-beds, and other similar porous articles, *wood*, such as plank, boxes, ships, wooden houses, &c., retain the greatest amount of *latent infection.* Hence yellow fever prevails most malignantly among crowded wooden buildings,‡ board-fences, temporary sheds, wharf-boats, &c., all of which retain the infection until it is neutralised by cold or frost.

---

* See Townsend on Yellow Fever of New York, pp. 252, 253.

† Idem, p. 116.—See Dr. Rush's Inquiries.

‡ The following fact stated by Dr. Bayley, in relation to the tenacity with which fætor from putrescent animal matters adheres to the wood and timbers of ships, will in some degree aid in illustrating the tenacity with which yellow-fever infection adheres to wooden houses, boxes, &c., viz:

"I have often noticed that those vessels loaded with hides and jerked beef which was partly damaged, and where the timbers and ceiling of the vessels became tainted, especially in the hold, the offensive smell has often been removed for the time, by being well ventilated and scrubbed with water:—but afterwards, upon the application of whitewash to the planks, *the offensive smell has been reproduced to a much greater degree than it was at first.* A second white-washing with lime, after the foul smell had again disappeared, again *renewed the putrid effluvia.* This would be produced in a decreasing degree for several times until the vessel was entirely purified." See Townsend on Yellow Fever of New York, pp. 94, 285–321.

This effect was produced by the chemical action of the lime, disengaging the putrid matter absorbed by the wood in a latent state. In the case of the brig Enterprise before cited, infection seems to have been absorbed by her timbers in the same way. The same action may be exerted upon the infection of yellow fever.

9. That yellow fever is a peculiar disease; a fever of only one paroxysm, varying from one to three days before the remission and collapse; and that it is radically different in its character from the whole family of remittent and intermittent fevers.

### YELLOW FEVER HAS BEEN EPIDEMIC IN THE FOLLOWING TOWNS AND PORTS OF THE UNITED STATES

1. Charleston, S. C. This is one of the oldest commercial ports in the United States, and has at all times had an extensive commercial intercourse with the West Indies; and it has likewise been subject to epidemic yellow fever repeatedly during its commercial prosperity.

The *first epidemic* was in the year 1700, again in the years 1703, 1728, 1745, 1748, 1753, 1755. The next was in 1793. From the year 1793 to that of 1807 inclusive, this disease prevailed in Charleston almost every alternate year, either severely or partially.* From that time until the restoration of commerce, after the peace of 1815, this city, as well as all other ports of the United States, was exempt from this disease. In 1817 the city was severely visited again; and from that time up to the year 1839, a period of 22 years of active and uninterrupted commerce, it has been epidemic in Charleston nine times, or nearly every alternate year. In this city the disease, always appearing among the vessels in port, and in unacclimated persons, is known chiefly by the name of the *stranger's fever.*

2. Philadelphia. This is one of the oldest commercial ports in the Union, but being in a more northern latitude was less liable to epidemic yellow fever than Charleston; yet it was occasionally visited by this disease up to the year 1762. From that time, during all the difficulties and interruptions of foreign commerce, until the close of the war of independence,

* See Med. Repos., old series, vol. ii, pp. 234, 235, and vol. iv, p. 100. See Dr. Simonds on Yellow Fever of Charleston.

the city was free from this disease. After the peace of 1783 the foreign commerce gradually revived, and having increased rapidly, Philadelphia became an important port, as the seat of the Federal Government. The consequence was, that yellow fever was introduced every alternate year, until the year 1808, when commerce was again interrupted by new difficulties with England and other European belligerants. This interruption, with the war which followed, gave Philadelphia another exemption for ten years. Since the year 1822, the city, having adopted the policy of New York in prohibiting infected vessels from the port, has enjoyed an exemption from these epidemics, and her commercial interests have been benefited rather than injured.

We may remark, that during the revolutionary war, the autumn of 1778 was *unusually sickly* along the whole Atlantic seaboard, the summer "*being unusually hot and sultry*": but there was no yellow fever, for the want of foreign infection, the general character of the diseases being of the "intermittent" type.* Yet so soon as commerce with the West Indies was fully revived, the diseases in the seaports dropped the "intermittent" character, and assumed that of epidemic yellow fever. During this time the British army and navy in the West Indies were annually ravaged by this pestilence which prevailed in nearly all the West India ports;† while the inhabitants of the interior were exempt.

3. New York, lat. 40° 43′ north. This city has been gradually increasing in point of population and commerce for the last fifty years, during which it has more than doubled its commerce and population. In a latitude almost too far north for epidemic yellow fever, the city has never been so extensively ravaged by this disease as the more southern ports. Yet it has suffered to a considerable extent, several times previous to the year 1808. After the great expansion of commerce after the late war, yellow fever was occasionally intro-

---

* See Med. Repos., old series, vol. ii, p. 364.
† Coxe's Med. Museum, 1805, vol. i, p. 184.

duced by vessels from the West Indies. But it did not spread epidemically except in 1819 and 1822, the latter being much the most fatal of any previous year.*

Since the year 1822, a period of nearly twenty years, a judicious quarantine has protected the city from epidemic yellow fever, although the commerce of the city during that time has been more extensive than it ever was previously; while Charleston, Savannah, Mobile, and New Orleans, without any quarantine restrictions, have suffered severely.

4. Savannah. This also is one of the oldest ports of entry. From a very early period it has been occasionally visited by epidemic yellow fever, when it had been prevailing extensively in the West Indies. From the year 1808 to 1817, during the interruption of commerce, it was exempt. Since that time it rarely escapes when Charleston, Mobile and New Orleans are visited. No quarantine regulations have been adopted in this city.

5. St. Augustine & Pensacola, under the Spanish occupancy, were proverbial for their salubrity, and yellow fever was unknown in them as an epidemic until the Floridas fell under the jurisdiction of the United States on the 17th of June, 1821.† As the treaty of cession had been made several months previously, the emigrants from the United States were pouring into St. Augustine early in the summer of 1821, while the Spaniards were departing as fast as possible for Cuba. Thus a constant intercourse was kept up between Cuba and St. Augustine, by numerous vessels which were transporting the Spaniards and their effects to Havana. These vessels on their return were chiefly freighted with tropical fruits, which were the means, no doubt, of producing an epidemic bilious fever, while some persons contracted yellow fever in the vessels direct from Havana. The following year, 1822, the American emigrants began to crowd into Pensacola; while transports were constantly passing and repassing to Havana, in removing the

*See Townsend on Yellow Fever of New York, passim A. D. 1822—also N. Y. Med. Repos., vol. ii, pp. 315 and 316.

† William's Florida, p. 207, &c.

persons and effects of the loyal Spaniards. The result was the introduction of a malignant epidemic, which spread rapidly among the unacclimated and promiscuous population who remained in the place. Again, in the autumn of 1825, the population was composed chiefly of recent northern emigrants, who were pouring in daily; and a brisk trade having sprung up with the port of Havana, the yellow fever was again introduced with great mortality. Since that period Mobile and other ports in the Territory of Florida, having withdrawn the trade from Pensacola, have likewise taken away the liability to frequent yellow fever epidemics; and for many years past, Pensacola has rarely suffered from yellow fever, although cases have been occasionally introduced in the national vessels of the United States and other powers.

6. New Orleans. Site on adhesive alluvial earth, or mud, 10½ feet above tide level, in lat. 29° 57′ north. This city has carried on a constant commercial intercourse with Havana and other West India ports, for more than seventy-five years, including the Spanish régime previous to 1803. During this long period, we are not able to ascertain that it has been visited by any malignant epidemic, until it came under the jurisdiction of the United States. Since that time, and especially since the revival of commerce, after the peace of 1815, its population and commerce have increased more rapidly than those of any city in the United States; and the frequency and mortality of yellow fever epidemics have been *pari passu.* In the period of twenty years from 1817 to 1837, the yellow fever has prevailed as an epidemic about nine times; and to such a degree of virulence that in 1819, when the resident summer population was only *thirty-three thousand souls,* the deaths in August were 560 souls, and in September 594. The yellow fever generally, if not invariably, begins its ravages among the shipping and in the population near the wharves.

We infer that any interruption to the commerce of New-Orleans and other Atlantic ports, such as that preceding and during the last war with Great Britain, would again suspend the prevalence of yellow fever in our ports.

For many years after the close of the war of independence, yellow fever was introduced occasionally into some other ports of less note than those we have already named. Thus it was introduced into Baltimore, into Wilmington, Delaware; Port Elizabeth, New Jersey; Norfolk, Virginia; Wilmington, Newbern, and Washington, North Carolina; New London, Connecticut, and Portsmouth, New Hampshire. Yet in most of these places, which were then important ports of entry, the disease manifested itself by only a few cases; and in those only, in most instances, who had had direct communication with infected ships, then lying in port,* which were charged with "foul air," as it was termed.

7. Gibralter and Cadiz, ports of Spain, have been visited by yellow fever. It prevailed in Gibralter in the summer of the years 1804, 1810, and 1813, and at Cadiz in the autumn of the year 1814. In each of these places it was introduced by vessels from the Spanish Main and the West Indies; and especially by the arrival of large fleets and armaments during the prevalence of the European wars against the power of Napoleon. It has also occasionally appeared in other ports of southern Europe at divers times between the years 1800 and 1815; but there are the strongest reasons to convince us that the disease was introduced by vessels and transports from the West Indies, during the wars which devastated Europe and the West Indies within that period. The troops and marines were those who suffered first and most severely.

